# Relationship between ABO blood group and von Willebrand factor levels: from biology to clinical implications

**DOI:** 10.1186/1477-9560-5-14

**Published:** 2007-09-25

**Authors:** Massimo Franchini, Franco Capra, Giovanni Targher, Martina Montagnana, Giuseppe Lippi

**Affiliations:** 1 Servizio di Immunoematologia e Trasfusione – Centro Emofilia, Azienda Ospedaliera di Verona, Verona, Italy; 2 Medicina Interna C, Dipartimento di Scienze Biomediche e Chirurgiche, Università di Verona, Verona, Italy; 3 Sezione di Endocrinologia e Malattie del Metabolismo, Dipartimento di Scienze Biomediche e Chirurgiche, Università di Verona, Verona, Italy; 4 Istituto di Chimica e Microscopia Clinica, Dipartimento di Scienze Biomediche e Morfologiche, Università di Verona, Verona, Italy

## Abstract

Although a number of studies have demonstrated the influence of ABO blood group on plasma levels of von Willebrand factor (VWF), the nature of this association and its clinical importance is still largely unknown.

In this review, the most recent advances in our understanding of the mechanisms by which ABO blood group determines plasma VWF levels and their clinical impact will be discussed.

## Review

### Introduction

Von Willebrand factor (VWF) is a large adhesive glycoprotein synthesized by endothelial cells and megakaryocytes that circulates in the plasma as a series of heterogeneous multimers [1-3].

VWF has two major functions in hemostasis. First, it is essential for platelet-subendothelium adhesion and platelet-to-platelet interactions as well as platelet aggregation in vessels in which rapid blood flow results in elevated shear stress. Second, VWF is the specific carrier of factor VIII (FVIII) in plasma and protects it from proteolytic degradation, prolonging its half-life in circulation and efficiently localizing it at the site of vascular injury [4].

While a deficiency of VWF is responsible for a hemorrhagic diathesis (von Willebrand disease, VWD) [5], there are increasing evidences that elevated VWF levels represent an important thrombotic risk factor [6,7].

Besides the *VWF *gene (12p12), it is well established that other gene loci exert major quantitative effects on VWF plasma levels. The most important of these loci has been shown to be the ABO blood group locus on chromosome 9q34 [8,9].

The antigens of the ABO system (*A*, *B*, and *H *determinants, respectively) consist of complex carbohydrate molecules. The *A *and *B *alleles encode slightly different glycosyltransferases that add N-acetylgalactosamine and D-galactose, respectively, to a common precursor side chain, the *H *determinant, converting it into *A*- or *B*-antigens. The *O *alleles do not encode a functional enzyme and consequently *OO *carriers, who lack such transferase enzymes, continue to express the basic, unmodified, *H-*antigen with a fucose moiety attached to precursor oligosaccharide chains[10].

The nature of the interaction between ABO blood group antigens and plasma VWF levels along with its clinical implication will be discussed in this review.

### Studies on the influence of ABO blood groups on VWF levels

Several studies have documented the influence of ABO blood groups on plasma VWF levels [11-13]. In a large twin study, Orstavik and colleagues [14] found that 66 percent of the total variation in plasma VWF levels was genetically determined and that 30 percent of this genetic component was explained by ABO blood group. Other studies have consistently reported that group O subjects have lower plasma VWF levels than non-O individuals. Indeed, in a large study of 1117 healthy individuals conducted by Gill and colleagues [15], plasma VWF levels were lowest in group O (mean von Willebrand factor antigen [VWF:Ag] 75 IU/dL) and highest in group AB subjects (mean VWF:Ag 123 IU/dL). From the same study also emerged that the influence of ABO blood groups on plasma VWF levels can make difficult the diagnosis of type 1 VWD since the normal range for VWF:Ag in group O individuals extends below 50 IU/dL (mean ± 2SD: 36–157 IU/dL), which is commonly accepted as the lower normal limit. In fact the authors, investigating the distribution of ABO blood groups in 114 patients with type 1 VWD, found a predominance of blood group O with a frequency significantly different from the general population (77% group O, 18% group A, 4% group B in type 1 VWD patients versus 45% group O, 45% group A, 7% group B in healthy individuals, P < 0.001) [15]. However, the primary importance of the bleeding history in the clinical decision to diagnose and treat type 1 VWD was stressed by Nitu-Whalley and colleagues in a retrospective study on the re-classification of 246 previously diagnosed type 1 VWD patients [16].

In addition to the above mentioned VWF antigenic variability, Moeller and colleagues [17] described variations in VWF activity (von Willebrand factor ristocetin cofactor, VWF:RCo) in persons with different ABO groups with individuals of group O that exhibited VWF:RCo levels significantly lower than those in the non-O group.

Miller and colleagues [18] studied the effect of ABO blood type and race on plasma VWF levels and found that Caucasians had significantly lower levels than African-Americans. Interestingly, ABO and race showed independently effects accounting for 19 and 7 percent of the total variance in VWF:Ag levels, respectively.

Besides the studies on ABO phenotype, other investigators have examined the relationship between ABO genotype and plasma VWF levels [19-21]. According with previous studies, lowest plasma VWF levels were present in genotype *OO *individuals. However, all groups also found that individuals heterozygous for the *O *allele (genotypes *AO*, *BO*) had significantly lower plasma VWF levels then those not carrying an *O *allele (genotypes *AA*, *AB*, BB).

Finally, other investigators studied the influence of Secretor blood group, characterized by a fucose as terminal sugar, on plasma VWF levels and found significantly higher VWF levels in individuals homozygous for the *Se *allele (genotype *SeSe*) compared to those heterozygous (genotype *Sese*) [22].

### The nature of the association

Von Willebrand factor is one of the few non-erythrocytic proteins that express ABO antigens. ABH oligosaccaride structures have been identified on the N-linked oligosaccharide chains of VWF located in the A1 domain, which contains the binding site for platelet glycoprotein Ib (GPIb) I [23]. The importance of VWF glycosilation in mediating the effect of ABO blood group on VWF levels is well demonstrated by the observation that aberrant endothelial expression of a glycosyltranferase (N-acetylgalactosaminyltranferase) results in altered VWF glycosilation and heightened clearance in mice [24]. O'Donnell and colleagues found a direct relationship between ABO genotype, A transferase expression and the amount of A antigen expressed on circulating VWF [25]. Similarly, Morelli and colleagues [26] demonstrated an allele specific, dosage dependent effect of the ABO alleles on VWF and FVIII levels and on the degree to which VWF was loaded with A and B antigens. Furthermore, A and B antigens expressed on VWF explained about 18 percent of the variation in plasma VFW levels.

The mechanism by which ABH determinants on plasma VWF influence plasma VWF levels is still unknown and several hypotheses have been made during the last years [9]. While the *in vitro *removal of A and B antigens from purified plasma VWF decreases VWF activity but not its antigenic level or binding to collagen [27], the detection of lower levels of all three in type O individuals suggests that ABO antigens *in vivo *influence the rate of synthesis or proteolysis/clearance of the whole VWF molecule rather than its function or multimeric structure [17].

The hypothesis that ABO blood group influences the rate of biosynthesis/secretion of VWF in endothelial cells is difficult to study, as glycan structures of VWF synthesized *in vitro *differ significantly from those of plasma VWF [28]. Furthermore, in vivo data do not support this theory as the total increase in VWF after infusion of desmopressin was not significantly different between group O and non-O individuals [29].

However, a more probable explanation for reduced VWF levels in blood group O individuals involves a decreased survival.

A central role for VWF glycan in determining the rate of hepatic clearance has been demonstrated in both animal models and humans. Changes in the glycosylation pattern of VWF in a mouse strain with altered expression of an N-acetylgalactosaminyltransferase gene resulted in low VWF levels, possibly because of an accelerated clearance [24]. Another direct link between VWF glycosylation and clearance comes from the study by Ellies and colleagues [30] on mice with absence of the enzyme ST3Gal-IV, which mediates the attachment of sialyl groups to terminal galactose residues. In ST3Gal-IV-deficient mice, the half-life of endogenous VWF was reduced two-fold. Similar results were observed by Sodetz and colleagues in rabbits [31]. A similar association between reduced ST3Gal-IV-mediated sialylation and reduced plasma VWF levels was also found in humans [31]. These data indicate that sialylation is important to prevent premature clearance via receptors that recognize non-sialylated terminal galactose residues, like the hepatic asialoglycoprotein receptor. Nossent and colleagues [32] used a mathematical approach and estimated that half-life of endogenous VWF is two hours longer than in the non-O population. Additional evidence that blood group O determinants on VWF are associated with increased clearance is provided by observations that infused FVIII (either plasma-derived or recombinant) disappears more rapidly in blood group O than in non-group O hemophilia A patients [33]. As regards the mechanism by which ABH structures on VWF may influence clearance rates, O'Donnell et al. originally proposed that clearance of VWF is mediated via the *H-*antigen by hepatic receptors specific for fucose exposing glycoproteins [25]. However, the subsequent finding that subjects with the Bombay phenotype, who totally lack *H*-antigen on VWF and are non-secretors (no H substance in plasma), have even lower VWF levels than blood group OO carriers seems to be in conflict with this model [34].

Recent studies have suggested that ABO blood group determinants may be important in influencing the susceptibility of plasma VWF to proteolysis by ADAMTS13 (a disintegrin and metalloproteinase with thrombospondin type-1 repeats-13) metalloprotease [9]. Bowen [35] purified VWF from individuals of different ABO blood groups and then incubated with human plasma-derived ADAMTS13. Mulimeric analysis and collagen-binding assays both demonstrated that proteolysis was significantly faster for group O VWF compared to non-O VWF. Thus, while carbohydrate structure has previously been shown to protect VWF from proteolytic degradation in plasma [36], the recent literature data show that a reduction in the number of sugars on the oligosaccharide chains of VWF is associated with an increased susceptibility to cleavage by ADAMTS13 [9]. Indeed, Bombay phenotype was found to be associated with an increased susceptibility to ADAMTS13 proteolysis [34]. The pathogenic mechanism by which ABO blood group influences the susceptibility to cleavage by ADAMTS13 is still unknown but two N-linked potential glycosilation sites (asparagines 1515 and 1574) are located in close proximity to the ADAMTS13 cleavage site (Tyr1605-Met1606 bond within the A2 domain). Thus, the oligosaccharide chain composition may be involved in stabilizing the conformation of this VWF region, such that the removal of terminal sugar allows the A2 domain to adopt a conformation more permissive for ADAMTS13 cleavage.

### Clinical implications

A number of studies have demonstrated a relationship between ABO blood group and hemostasis. Indeed, a higher rate of bleeding complications has been described in patients belonging to group O [37,38] and blood group O individuals are consistently overrepresented in patients with inherited bleeding disorders [15]. By contrast, an association between non-O blood groups and the risk of arterial thromboembolic disease, including ischemic heart disease and peripheral vascular disease, has been recognised by several studies [39-42]. Results from the Hoorn study [43] indicated that blood group non-O was associated with a twofold increased cardiovascular mortality compared with blood group O (RR 2.08 [95% CI 0.85–5.07]). The increased risk of venous thromboembolism in non-O individuals has also been reported [44-47]. Interestingly, Morelli and colleagues [48] found that carriers of non-O alleles had a twofold increased risk of a first deep vein thrombosis and that the non-*OO *genotypes strongly influenced the risk of thrombosis in FV Leiden carriers. On the other hand, both arterial and venous thrombotic risks have been related to VWF levels [49-51]. However, only few studies have examined the effect of VWF levels on the association between ABO blood type and thrombosis.

To elucidate the role of the ABO blood group, VWF and FVIII in the process of deep vein thrombosis, a population-based patient-control study on 301 consecutive patients and 301 controls was performed by Koster and colleagues [49]. In univariate analysis the authors found that the risk of thrombosis increased with increasing VWF and FVIII levels and was higher in non-O subjects than in those of group O. However, in multivariate analysis only FVIII remained as a risk factor, suggesting a close relationship between blood group and VWF concentration.

Tirado and colleagues [52] analyzed 250 patients with venous thrombosis and 250 unrelated controls and found higher VWF levels in non-O group, which was more frequent in patients. However, the risk attributed to VWF was strongly dependent on blood groups as it disappeared after adjusting for the ABO groups. Similar results were found by Ohira and colleagues [53] in the Longitudinal Investigation of Thromboembolism Etiology (LITE) study.

On the whole, the results of these studies indicate that, compared with type O, non-O individuals could have an increased thrombotic risk via having higher VWF-FVIII levels. However, not all studies agree with these findings as some authors found that the association between VWF and the risk of cardiovascular mortality was independent of blood group [40,43].

## Conclusion

A number of studies demonstrates that ABO blood group influences plasma levels of VWF. Recent investigations seem to indicate an altered susceptibility to cleavage by ADAMTS13 as an important mechanism by which ABO group can affect the rate of VWF catabolism. However, the elucidation of the physiology of glycan structures of VWF will help to understand the mechanisms by which blood group contribute to the thrombotic risk.
